# Between form and function: the complexity of genome folding 

**DOI:** 10.1093/hmg/ddx306

**Published:** 2017-08-16

**Authors:** A. Marieke Oudelaar, Lars L.P. Hanssen, Ross C. Hardison, Mira T. Kassouf, Jim R. Hughes, Douglas R. Higgs

**Affiliations:** 1 MRC Molecular Haematology Unit, Weatherall Institute of Molecular Medicine, University of Oxford, OX3 9DS, UK; 2 Department of Biochemistry and Molecular Biology, Pennsylvania State University, University Park, PA 16802, USA

## Abstract

It has been known for over a century that chromatin is not randomly distributed within the nucleus. However, the question of how DNA is folded and the influence of such folding on nuclear processes remain topics of intensive current research. A longstanding, unanswered question is whether nuclear organization is simply a reflection of nuclear processes such as transcription and replication, or whether chromatin is folded by independent mechanisms and this *per se* encodes function? Evidence is emerging that both may be true. Here, using the α-globin gene cluster as an illustrative model, we provide an overview of the most recent insights into the layers of genome organization across different scales and how this relates to gene activity.

## Introduction

Nuclear organization is currently described at vastly different scales. Many nuclear structures, such as euchromatin, heterochromatin, the nuclear lamina, and non-membrane bound nuclear sub-compartments, can be identified by conventional microscopy. Technological advances have revealed finer detail of nuclear structure, including chromosome territories and the organization of telomeres and centromeres. More recently, sub-microscopic features of nuclear structure such as chromatin folding, chromatin structure, and distribution of chromatin-associated proteins have been determined, and many of these features can now be correlated with specific DNA sequences (1,2). An important aim of current research is to relate these diverse observations, at different levels of resolution, to each other and to obtain an integrated understanding of the relationship between nuclear organization and function. This is likely to provide important insights into how mutations affecting various aspects of nuclear organization may cause human genetic disease.

A key question in this field, discussed here, is whether sub-chromosomal organization is predominantly an emergent property of a wide variety of nuclear processes, or established independently, representing ‘building blocks’ of the genome.

## Self-Interacting Domains of Chromatin

It has previously been shown that individual eukaryotic chromosomes fold in such a way that they more frequently interact with themselves rather than with other chromosomes and occupy distinct chromosome territories in the nucleus (3). Analyses at higher resolution using chromosome conformation capture (3C) techniques (4,5) have suggested that individual chromosomes may be further organized into a variety of self-interacting domains.

**Figure 1:**
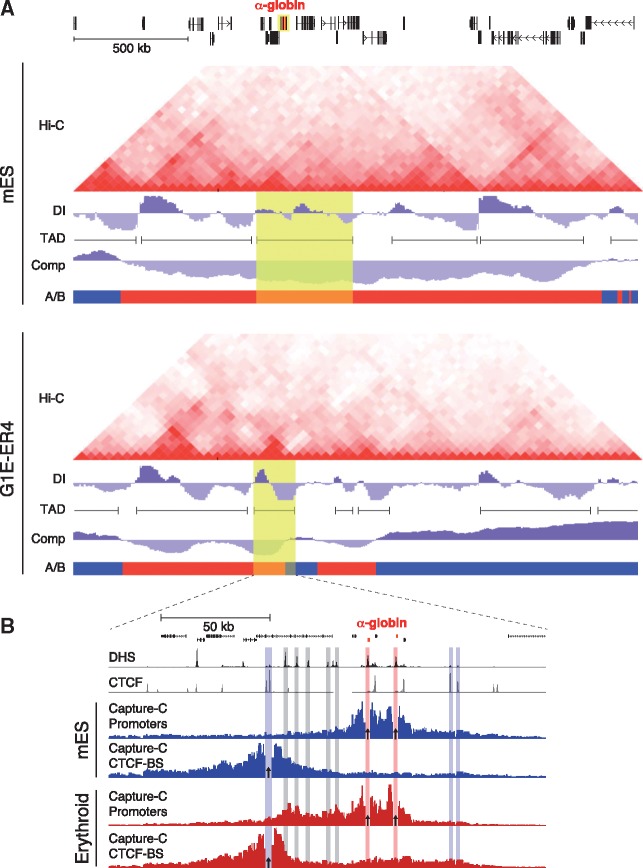
The structural organization of the murine α-globin cluster in its inactive and active conformation. (**A**) Structural features of the α-globin cluster (2.5 Mb) defined by Hi-C in inactive mES cells and G1E-ER4 erythroid progenitor cells with basal α-globin transcription. The adult α-globin genes (*Hba-1* and *Hba-2*; shown in red) are located in a gene-rich region of the genome shown at the top. The Hi-C heat maps underneath display interaction frequencies in mES cells and G1E-ER4 cells at 40 kb resolution (64,65). The tracks below each heat map display the directionality index (DI), from which the TAD calls shown are derived. Compartment scores (Comp) and the separation into compartment A (active; shown in red) or B (inactive; shown in blue) are displayed at the bottom. The TADs containing the α-globin cluster are highlighted in yellow. The Hi-C heat map and derived features were generated using resources from the VISION project (http://www.bx.psu.edu/∼giardine/vision/), including the 3D browser (66). (**B**) Detailed interaction data describing the α-globin locus (200 kb) at high-resolution using Capture-C data in inactive mES cells and active primary erythroid cells. The α-globin genes are shown in red at the top, followed by tracks displaying DNaseI Hypersensitivity Sites (DHS) and CTCF occupancy in primary erythroid cells. Capture-C interaction profiles generated from the viewpoint (indicated by arrows) of the duplicated α-globin promoters and an upstream CTCF-binding site (CTCF-BS) that forms one of the domain boundaries, are shown below (35,54).

One of the first classes of self-interacting domains described relatively large regions of chromatin (100–5 Mb) observed in Hi-C heat maps, which were named topologically associated domains (TADs) (6,7). TADs were originally defined by the preferential interaction of regions located within the same domain and the relative depletion of interactions between regions located in adjacent domains. TAD boundaries were initially identified using a directionality index (DI) (6), which quantifies the degree of upstream or downstream interaction bias for a genomic region. Several other algorithms for TAD identification have been developed, and it has been shown that the number of TADs called can vary depending on the algorithms used, the thresholds applied, and the resolution of the underlying data (8). Nevertheless, many TADs correlate with chromatin signatures, gene activity and replication timing (7,9,10). Based on Hi-C heat maps at relatively low resolution, it has been suggested that TADs are relatively stable across different cell types and conserved across species. At present TADs are the most frequently described self-interacting domains. Typical examples are shown in mouse embryonic stem (mES) cells in Figure 1A.

Similar Hi-C data have also been interpreted from a different perspective that describes larger structures called compartments. These are composed of transcriptionally active or inactive TAD-like structures which cluster together to form functionally distinct nuclear compartments (11,12). The active TAD-like regions (compartment A) are thought to form higher-order structures in the interior of the nucleus; the inactive TAD-like regions (compartment B) are located closer to the periphery. It has been shown that lamina-associated domains (LADs), which have been associated with gene repression, largely correspond to compartment B (2,13).

More recent analyses of nuclear compartments and TADs using a variety of 3C techniques have revealed previously unrecognized, smaller, self-interacting domains that have been referred to as contact domains (12), sub-TADs (14), insulated neighborhoods (15) and frequently interacting regions (16).

A common feature of all self-interacting domains, is that their borders are enriched for boundary elements that among many other proteins commonly bind CTCF, a highly conserved DNA-binding zinc finger protein. CTCF is strongly implicated in the formation of self-interacting domains, which often depends on the presence of pairs of CTCF-binding motifs in convergent orientations at the domain borders (12,17–21).

The definitions of the structures discussed above are rather vague and their identification somewhat arbitrary (8). The described domains are all identified by their self-interacting features, boundary elements, and sizes. To an extent, self-interacting domains appear to be fractal structures in the genome; the definition of each unit depending on the level of resolution. It is therefore unclear if they represent fundamentally different structures, or if they are simply variants of a similar organizational process.

## The Formation of Self-Interacting Domains

It has been proposed that self-interacting domains are formed by a process of loop extrusion. This is thought to be an active process in which a loop-extruding factor, containing two DNA-binding units, associates with chromatin and travels along the fiber in opposite directions, creating a progressively larger intervening loop, until the factor is stalled at boundary elements, such as convergent CTCF-binding motifs. Hetero-dimerization between CTCF proteins or other boundary proteins then tethers the extrusion factor, defining the limits of the self-interacting domain (20,22). It can be predicted that during the dynamic formation of such loops in a population of cells, every region within a self-interacting domain, at some point, contacts every other region lying between the border elements. This is consistent with the increased interaction frequencies observed between all genomic regions within such domains either at low (Fig. 1A) or high resolution (Fig. 1B). Based on its co-localization with CTCF at domain boundaries, cohesin, a ring-shaped protein complex that contains ATPase activity and can interact with two double-stranded DNA helices, is thought to be a strong candidate for an extruding factor in the mammalian genome (23–28).

In support of the loop extrusion model, it has been shown in various studies that mutation or deletion of specific CTCF-binding sites can disrupt the border of the associated self-interacting domain, and cause a change in genome structure that allows enhancers within the domain to interact with genes in neighboring domains, resulting in mis-regulated gene expression (7,15,17,29–35). This supports the important role of CTCF/cohesin boundaries in delimiting self-interacting domains, but as these proteins are ubiquitously expressed and widely bound to chromatin, they alone cannot explain the tissue-specific structures observed in some of these studies (30,35). This is clear in the murine α-globin locus shown in Figure 1B, which is flanked by two double CTCF-binding sites, which are occupied by CTCF and cohesin in both erythroid and non-erythroid (mES) cells, but only interact in erythroid cells.

A possible explanation could be that cohesin is recruited to chromatin in a tissue-specific manner. It has been shown that cohesin is not only associated with CTCF-binding sites, but also with active promoters and enhancers and that dynamic cohesin occupancy of enhancers correlates with tissue-specific interactions between promoters and enhancers (36–38). Furthermore, it has been shown that active transcription is involved in distributing cohesin along the genome (39).

It is important to keep in mind that the self-interacting domains observed in 3C analyses represent a population average of a dynamic structure and that the spatial separation between TADs is not absolute. Insulation between domains does not completely prevent the formation of contacts across boundaries (12) and such interactions can have functional effects on gene expression (40). These long-range interactions are infrequent and therefore likely to be highly dynamic. Single-cell Hi-C studies have identified TADs in individual cells, but have also shown that the level of compaction within TADs is variable and that their higher-order organization in larger structures (e.g. compartments) is stochastic (41,42), which can be explained from the assumed polymeric behavior of chromosomes (10).

## The Conservation of Self-Interacting Domains Across Species, Tissues and Developmental Stages

Comparisons between mouse and human Hi-C maps from ES and B-lymphoblast cells have shown that approximately half of the identified large self-interacting domains in orthologous regions are conserved (9,12). A similar conservation of chromosome structure was observed in a study in which Hi-C maps from hepatocytes isolated from four mammalian species were examined (18). Furthermore, these self-interacting domains have been shown to be similar in different tissues. Comparisons between cell types have shown that approximately 50–60% of TADs (6,12,16,43) and approximately 40% of the functionally distinct A/B compartments (16) are invariant. In some areas of the murine genome, self-interacting domains are thought to encompass regulatory regions (enhancers and promoters) to form structures, early in development, that are found in all tissues and which are subsequently modified in a cell type-specific manner (44). A similar picture has been observed in Drosophila, where many long-range interactions are similar across tissues and developmental stages (45). Importantly, rearrangements in mouse and human leading to duplication or deletion of entire self-interacting domains at the *Sox9* locus have been shown to be less disruptive to gene expression and cause less extensive changes in phenotypes than those which partially disrupt a self-interacting domain (34).

These observations at specific loci provide preliminary evidence for a mechanism which organizes the genome early in development independently of gene expression and chromatin modification and yet plays an important role in regulating subsequent gene expression. The possibility that these structures form before gene activation would predict that specific factors, elements or processes are capable of generating a chromatin environment that promotes the formation of interactions between regulatory *cis-*elements (46). Mutations affecting such factors would alter chromatin structure and therefore gene regulation, while other mutations may only affect the ability of a *cis*-element to influence a promoter, without substantially changing the structure. Although CTCF, cohesin and their binding sites are strong candidates, *direct* experimental evidence supporting their role in forming such constitutive structures imposing a regulatory role in gene expression is limited, but under intense investigation.

Functional experiments have shed further light on the role of CTCF and cohesin in the formation of self-interacting domains and their effects on gene expression. A recent study showed that complete depletion of CTCF resulted in a loss of TAD structure, but left active and inactive genome compartments properly segregated (47). Surprisingly, the effect on gene expression appeared relatively modest. In contrast to the strong effects of CTCF on genome architecture, early studies of cohesin depletion showed only moderate changes (48–50). However, depletion in these studies was not complete, which probably confounded these conclusions. A more recent study used deletion of the cohesin loading factor *Nipbl* to efficiently displace cohesin from chromatin. This had a greater effect on gene expression and caused dramatic genome reorganization in which TADs vanished globally. The disappearance of TADs unmasked a finer compartment structure that reflected transcriptional activity and chromatin state and these domains remained segregated into compartments A and B (51).

## The Interplay Between Self-Interacting Domains, Nuclear Processes and Gene Activity

The observations described above suggest that CTCF and cohesin play a role at some, but by no means all, CTCF-binding *cis*-elements in establishing and/or maintaining self-interacting domains. However, the observation that some aspects of self-interacting domains persist in the absence of CTCF or cohesin, shows that these structures are more complex than first thought. This has also been suggested by computer simulations of polymer models (52,53). A recent study showed that models based on CTCF-mediated architecture alone could not predict *in vivo* genome organization. However, when interactions between other *cis*-elements were included in the models, the observed self-interacting structures could be better explained (52).

It seems therefore possible that there are at least two (largely) independent mechanisms underlying self-interacting domains. One might involve a process of loop extrusion that depends on CTCF, cohesin and/or other boundary associated proteins. This sets up constitutive architectural domains that are largely tissue-invariant and separate some regions of the genome into domains that restrict interactions between regulatory elements.

In addition, nuclear processes such as changes in chromatin accessibility, the binding of key transcription factors to *cis*-elements, increased enhancer-promoter interactions and active transcription may further shape the structure of these domains. Cohesin may also play a role in these interactions (37). The domains formed by these mechanisms are highly tissue-specific (54) and cluster together to form compartments with similar chromatin states and activities.

Although some of the processes driving constitutive and cell type-specific structures may be independent, they may nevertheless influence each other. For example, the constitutive structures thought to be set up by cohesin/CTCF may constrain interactions between regulatory elements and therefore contribute to their specificity (55–58). Furthermore, the recruitment and localization of cohesin in tissue-specific domains might be influenced by transcription factors, co-activators and the active process of transcription (37,39).

The structures related to nuclear processes were initially described as chromatin ‘loops’ formed specifically between active promoters, enhancers and boundaries (59). However, high-resolution, quantitative 3C data show that rather than specific peaks of interaction over these *cis*-elements, broad, self-interacting domains may appear over relatively large genomic regions upon activation of such elements in specific cell types. Within this region, increased interactions over certain elements can be identified (54,60) (Fig. 1). This is consistent with a mechanism such as loop extrusion promoting a general increase in self-interaction over a given genomic region delimited by boundary elements. Increased interactions over certain *cis*-elements suggest that when such elements, bound by multi-protein complexes, are brought together, they form more stable or frequent interactions than the rest of the self-interacting domain.

Though nuclear processes related to gene expression likely influence genome structure, current evidence argues against a role for transcription *per se* in establishing self-interacting domains: a recent study of chromatin conformation during Drosophila embryogenesis showed that the appearance of TADs occurs independently of transcription (61). However, this study also showed that although higher-order chromatin conformation and intra-TAD contacts are still present, albeit reduced, in embryos in which transcription was abolished, the lack of transcription nevertheless affects the properties of TAD organization and resulted in a significant loss of inter-TAD insulation.

**Figure 2:**
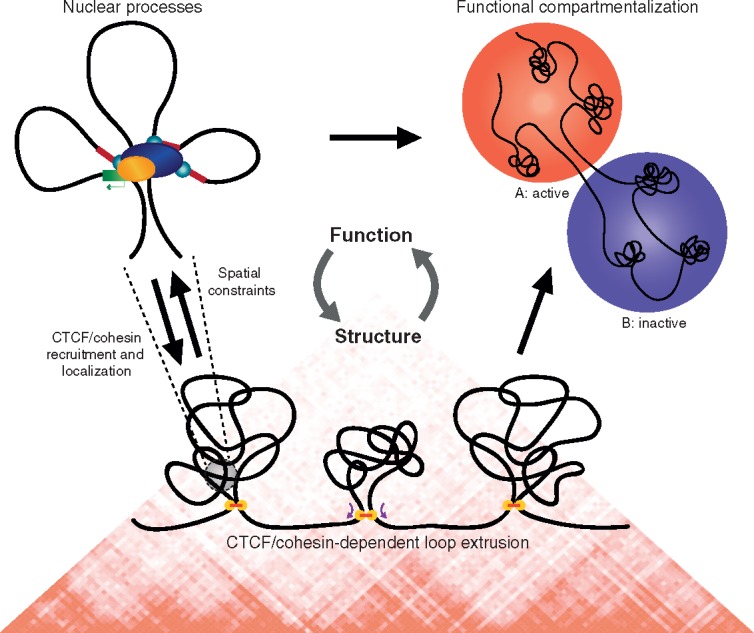
Proposed model for the processes contributing to chromatin structure and their interplay with genome function. TAD-like structures observed in Hi-C maps may be formed by a process of CTCF/cohesin-dependent loop extrusion (bottom). These domains restrain the connectivity between genes and regulatory elements, but are simultaneously influenced by the interactions between, and activity of, the elements they contain (left). The activity of these domains determines the higher-order organization of TAD-like structures into functionally distinct compartments inside the nucleus (right).

Overall, these studies suggest that the structures observed in contact matrices result from a mixture of different mechanisms and reflect an intricate interplay between form and function (Fig. 2).

## Analysis of Self-Interacting Domains Containing and Surrounding the α-Globin Cluster

The globin loci have been used extensively to establish general principles of mammalian genome organization and gene regulation and relate perturbations in these processes to human genetic disease. Principles first established at these loci have usually been found to be true of many other loci (62,63).

To examine the concepts discussed here, we analyzed 3C interaction data for the well-characterized murine α-globin cluster. Some self-interacting domains in this gene-dense 2.5 Mb region appear as relatively discrete structures in mES cells (Fig. 1A, top panel). However, other areas form poorly defined structures, and appear to be organized in a fractal pattern. This suggests that even though some areas of the genome are organized into well-defined, preformed, self-interacting domains, this by no means applies to the entire genome.

Figure 1 also shows a comparison between mES cells, in which the α-globin cluster is not expressed, and G1E-ER4 cells, in which α-globin is expressed at basal levels. This specific example clearly shows that the self-interacting domains, which in this case correspond to TADs, are dynamic, and quite different between these two cell types. Activation of the α-globin genes in G1E-ER4 cells is accompanied by increased interactions between promoters and enhancers, the appearance of smaller, more specific self-interacting domains, and a shift of regions adjacent to the active α-globin cluster from compartment A (active) to compartment B (inactive). This shows that not all self-interacting domains are conserved across tissues.

Comparison between primary erythroid and mES tissue at higher resolution (Fig. 1B) shows that activation of the α-globin genes and their *cis*-acting regulatory elements causes the formation of a strong self-interacting domain, in which contacts between the α-globin promoters and enhancers are significantly increased. The boundaries of this domain are formed by predominantly convergent CTCF-binding sites. These become closely apposed specifically in erythroid cells, while no interactions between convergent CTCF sites are observed in mES cells. This shows that this self-interacting domain is not preformed, but becomes established in a cell type-specific manner. It has recently been shown that these CTCF/cohesin boundaries constrain the activity of the α-globin enhancers to the self-interacting domain (35).

## Conclusions

Early studies of nuclear organization of the genome at relatively low resolution showed the existence of self-interacting domains, such as TADs, which were initially interpreted to be evolutionarily conserved and invariant between cell types. More recent analysis at higher resolution suggests that these domains may represent a fractal structure, with many domains contained within previously defined TADs and compartments. In addition, some of these domains appear to be tissue-specific.

Current data are consistent with at least two processes contributing to the observed nuclear organization. In some areas of the genome, preformed self-interacting domains may exist, which are formed by processes that are not fully understood, but likely depend on CTCF and cohesin. Processes related to gene expression within these domains impose further organization on the genome in a cell type-specific manner (Fig. 2).

If the current model is correct, there should be more examples of human genetic diseases that result from perturbations of the formation of self-interacting domains rather than from mutations of genes and their promoters or enhancers. Once the *cis*- and *trans-*acting factors responsible for establishing these domains have been identified, it should be possible to mutate these precisely and rigorously to demonstrate their role in nuclear organization and gene regulation.

To further understand the origins and effects of self-interacting domains, we require more high-resolution analyses, of individual examples, such as the α-globin cluster discussed here, where interactions between the constituent elements can be related to processes leading to gene expression.
